# Interfacial phenomenon and Marangoni convection of Fe–C melt on coke substrate under in situ observation

**DOI:** 10.1038/s41598-023-42631-y

**Published:** 2023-09-20

**Authors:** Lihua Gao, Yibin Huang, Wenlong Zhan, Chuan Wang, Zhijun He, Qinghai Pang, Junhong Zhang

**Affiliations:** 1 School of Materials and Metallurgy, University of Science and Technology Liaoning, Anshan, 114051 China; 2 Metallurgy Department, Swerim AB, 971 25 Luleå, Sweden; 3 Material Science and Engineering, KTH Royal Institute of Technology, 100 44 Stockholm, Sweden

**Keywords:** Chemical engineering, Theory and computation

## Abstract

The interfacial phenomenon between liqiuid iron and coke is important for determining the melting efficiency in the blast furnace iron-making process. In this study, the interaction observed in the case of the iron-carbon (Fe–C) melt on coke substrate was investigated using a high-temperature vacuum wettability test equipment. The Fe–C melt did not wet and spread on the coke substrate with different graphitization degrees (*r*_0_) at a high temperature of 1450 °C. The contact angles changed from 124.5° to 105.3°, and the *r*_0_ increased from 9.30 to 50.00%, thus indicating a nonwetting state. The deepening of graphitization decreased the contact angle. Thereby, increasing the contact area between liquid iron and the carbonaceous material, which facilitated carbon dissolution. The irregular movements of Fe–C melt were observed in situ during the wetting process. The horizontal force of the droplet caused by interfacial tension and the contact angle; the Marangoni convection owing to the gradient of carbon concentration; and the impulse force caused by the generation, aggregation, and release of SiO bubbles at the interface were attributed to the driving force.

## Introduction

Reduced carbon dioxide emissions and effective use of carbon resources have been the goals of iron and steel enterprises to achieve the objectives of satisfying the energy demand and environmental conservation since carbon consumption is the largest source of greenhouse gas emissions in the blast furnace (BF) iron-making process^1^.

The depletion of high-quality coking coal resources, high-grade iron ore, and the degradation of the environmental situation can be attributed to the extensive development of large-scale iron and steel industry. This necessitates the urgent requirement for low-carbon BF operation; however, pulverized coal or other substitute fuels cannot replace coke’s mechanical role in providing the permeability to the upward flowing gases in the BF^2–5^. The low-carbon BF operation may result in the thinning of the coke layer, which causes the instability of burden mixing, reduction in furnace permeability, gas channeling, and a delay in the reduction reaction, all of which considerably impact the smooth BF operation^6–10^. Furthermore, the coke layer thinning weakens the solid–liquid carburization process when liquid iron passes via the coke, which lowers the carbon concentration in hot metal and deepens the corrosion of the unsaturated liquid iron to the hearth refractory^11–15^. It is important to accelerate the carburization rate of liquid iron in the thin coke layer and improve the burden’s permeability to enhance the operational stability, the production efficiency, and ensure the longevity of BF.

Carbonaceous materials with a high crystal series stack height (*Lc*) have a high carbon dissolution rate owing to interdependence between the order of structure and carbon dissolution rate^16–20^. The dissociation of carbon atoms in graphite is faster than that of coke owing to the arrangement of carbon atoms in a 2-D array. Consquently, a high *Lc* value will help in the decomposition of carbon atoms. Khanna et al. discovered that the dissolution rate of these carbons is considerably slower than that of synthetic and natural graphite when investigating the carbon dissolution rate in various types of coal chars with low *Lc* values^21^**.** Xu et al. compared the dissolution characteristics of solid fuels (coke, semicoke, and lump coal) used in COREX gasifiers in liquid iron. They discovered that the reaction rate of coke, semicoke, and lump coal sequentially increases at the same temperature. The separation of carbon atoms from the carbon matrix is limited by the type of bond between the carbon atoms. The high graphitization degree (*r*_0_) of carbon structure and the low bond energy between its atoms facilitates the simple dissociation of carbon atoms from the matrix. The *Lc* value of lump coal, semicoke, and coke sequentially increases, and the carbon crystal structure approaches the ordered graphite structure, enabling a rapid dissolution rate of coke^22^. Previous studies primarily emphasized the carbon structure and carbon dissolution rate with few studies focusing on the Fe–C interface reaction and wetting mechanism at high temperatures.

Coke is a porous carbonaceous material in which the amorphous carbon structure gradually transforms to the regularly arranged graphite structure at high temperatures^23–25^**.** This study investigates the change in *r*_0_ of coke at 1200 °C, 1400 °C, and 1600 °C using X-ray diffraction (XRD). The wettability of coke substrates with varying *r*_0_ and Fe–C melt was examined using the improved sessile drop method and high-temperature vacuum wettability test equipment. Compared with similar conventional methods for measuring the wettability, the proposed method can be adopted under high temperature. The alloy sample is dropped onto the coke substrate once reaching the experimental temperature to avoid any reactions in advance. Then, the interface layer morphology and elemental distribution were examined using scanning electron microscopy (SEM) and energy-dispersive spectroscopy (EDS).

## Experimental

In the present paper, the coke sample was taken from a Chinese commercial blast furnace at Baosteel Company. The chemical analysis of the coke sample is shown in Table 1.

**Table 1:** Chemical analysis of the selected coke (mass fraction/%).

Ultimate analysis (dry basis)					Proximate analysis (dry basis)		
C_d_	H_d_	O_d_	N_d_	S_d_	Fixed carbon	Volatile	Ash
85.46	0.48	0.37	1.04	0.75	85.23	1.77	13.00

The large irregular coke was cut into 20 × 20 × 5 mm^3^ cuboid substrate using a disc cutter, and the graphitizing coke experiment was conducted using a tube resistance furnace with a programmable temperature control. The heat treatment temperatures of coke sample were set to 1200 °C, 1400 °C, and 1600 °C. The constant temperature was maintained for 2 h after reaching the aforementioned target temperature by cutting off the power and conducting the entire process under an Ar atmosphere. The prepared coke samples were crushed to fine powders of less than 74 μm in size. The XRD patterns were obtained by recording the scattering intensities of powder samples using a Rigaku diffractometer (Ultima IV, Rigaku Corporation, Tokyo) with Cu Ka radiation (40 kV, 40 mA) as the X-ray source; the scanning angles were in the range of 10 to 90 deg (2 h) at a scan rate of 20 deg/min. The Fe–C melt with 4.3% carbon content was then selected. To ensure uniform composition, an appropriate amount of high-purity Fe powder (99.99%, mass fraction) and graphite powder (99.99%, mass fraction) were weighed according to the set ratio, mixed with a ball mill, and smelted in a tubular resistance furnace. The melted Fe–C samples were cut into small cubes of 3 × 3 × 3 mm^3^ using a wire cutting technology. Before the experiment, the cut Fe–C melt samples and coke plates underwent ultrasonic cleaning three times in alcohol, followed by drying in an oven for 2 h.

**Figure 1:**
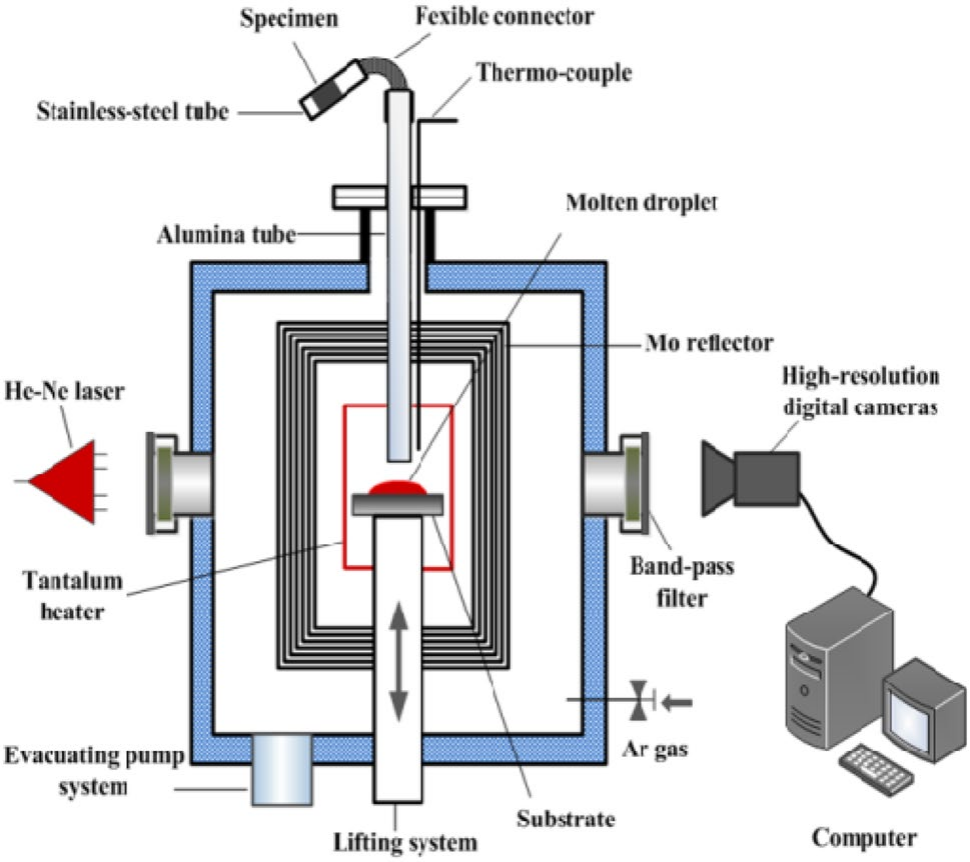
High-temperature vacuum wettability test system.

The improved sessile drop method was used to measure the wettability of Fe–4.3% C melt and coke substrates with varying *r*_0_ at 1450 °C in vacuum. Since the the tapping temperature of hot metal in the production reality of BF is between 1400 °C and 1500 °C, the appropriate temperature for wetting experiments was chosen as 1450 °C to simulate the actual temperature in BF. The experimental setup is shown in Fig. 1. First, place the cleaned coke substrate on the alumina base and adjust the base to a horizontal position. The sample was temporarily stored in a steel tube, which was maintained in vacuum at 25 °C under 5 × 10^–4^ Pa. After heating the equipment to 1450 °C, the alloy sample in the storage tube is dropped onto the coke substrate via the Al_2_O_3_ tube connected to the inside of the furnace cavity; the image is then captured with a high-resolution digital camera at the same time. The shooting stops when the droplet shape is unchanged.

After the wetting experiment, the Fe–C melt sample was allowed to cool in the furnace and the axisymmetric droplet shape analysis software was used to process images collected during the wetting process to determine the contact angle between Fe–C melt droplets and coke substrate. The surface morphology and elemental distribution of sample were analyzed using SEM and EDS.

## Results and discussion

### Graphitization test of coke

**Figure 2:**
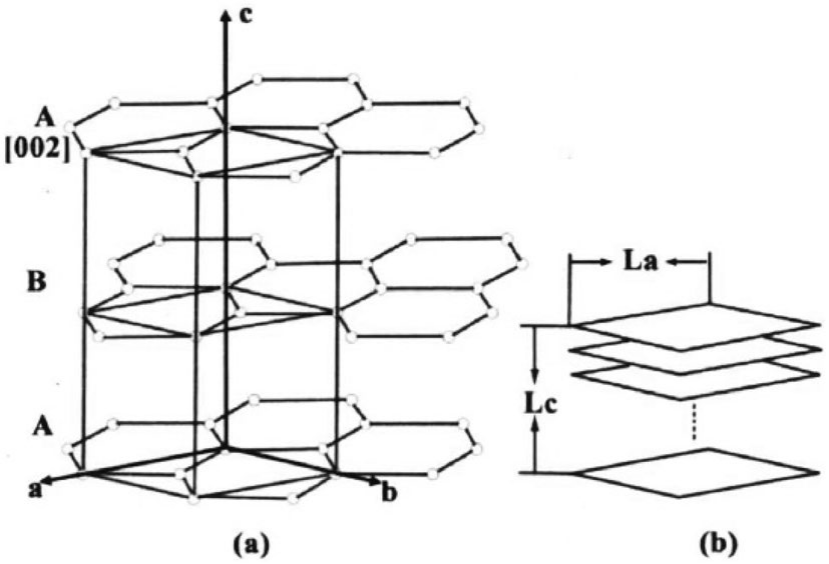
Schematic of graphite crystal structure (**a**) Graphite crystallite structural unit, (**b**) Correlation between crystallite and *La*, *Lc*.

Figure 2a shows the graphite carbon structure as a vertical regular hexagonal aromatic layer^26^. The crystallite size can be represented by the spread of carbon net plane (***La***) and the thickness of carbon net layer (***Lc***). Figure 2b illustrates the correlation of crystallite with ***La*** and ***Lc***.

The Scherrer equation^27^ can be used to calculate crystallite sizes such as the *Lc* and *La* values, respectively.

1 $$ L = \frac{k\lambda }{{\beta \cos \theta }}, $$

where, *k* is the constant for a given crystal plane (*L*_002_: *k* = 0.89, *L*_100_: *k* = 1.84), *λ* is the X-ray wavelength (1.5418 Å), *β* is the full width at half maximum (FWHM) of the X-ray diffraction peak, and *θ* is the Bragg diffraction angle. When calculating *Lc* and *La*, the diffraction peaks are selected along the (002) and (100) planes, respectively.

The average distance *d*_002_ between graphitic carbon layers can be calculated using the Bragg equation, as shown in Fig. 2b^28^:

2 $$ \lambda = 2d_{002} \sin \theta_{002} , $$

The FWHM method was used to measure the average distance *d*_002_ between the carbon layers of graphite; moreover, *r*_0_ can be calculated using Eq. (3):

3 $$ r_{0} = \frac{{\Delta_{d} }}{{\Delta_{0} }} = \left( {\frac{{3.44000 - d_{002} }}{0.08600}} \right) \times 100\% , $$

where, Δ_*d*_ (in Å) is the difference between the interlayer spacing of completely nongraphitized coke material and the interlayer spacing of the graphite material to be measured and Δ_0_ is the difference between the interlayer spacing of the completely nongraphitized coke material and the ideal graphite material (3.440 − 3.354 = 0.086) Å.

Unlike amorphous carbon in coke, which only produces the diffraction peak’s background, the crystalline graphitized carbon produces the XRD peak. Carbon diffraction peaks are ~ 25° and 45° on the (002) and (100) crystal planes, respectively.

**Figure 3:**
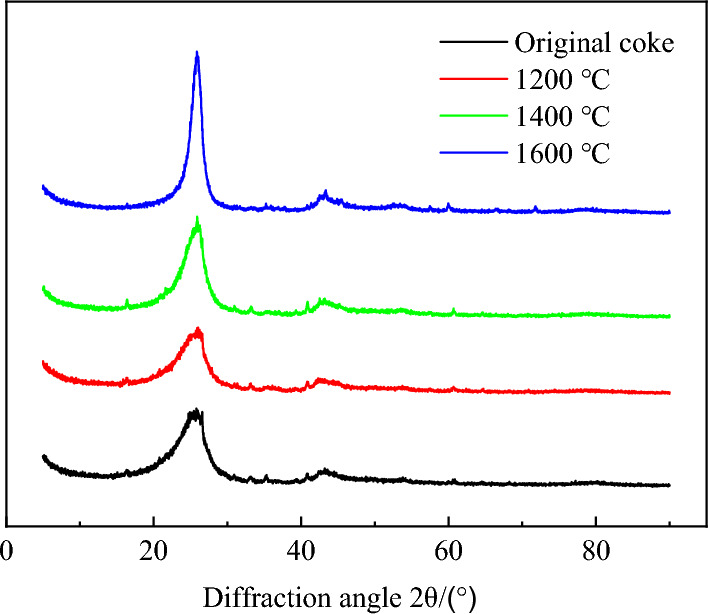
X-ray diffraction patterns of coke at different heat treatment temperatures.

Figure 3 shows the XRD pattern of coke at various heat treatment temperatures. The crystallinity or degree of ordering of carbon structure can be qualitatively assessed from the shape of the (002) carbon peak. A narrow peak represents a greater degree of ordering of carbon. Comparison of XRD patterns of the coke sample shows that the width of (002) carbon peaks became narrower with increasing temperature, indicating an increased degree of ordering of carbon layers.

**Figure 4:**
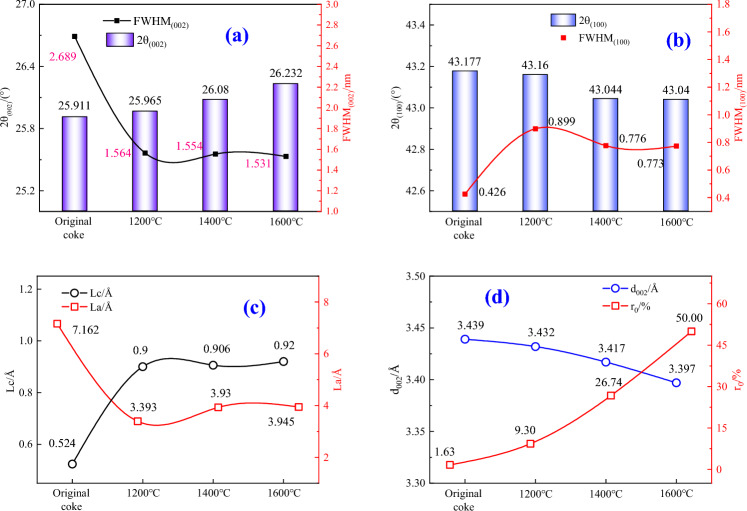
The XRD parameters of coke at different heat treatment temperatures. (**a**) The FWHM_(002)_ and 2θ_(002)_ values of different coke samples; (**b**) The FWHM_(100)_ and 2θ_(100)_ values of different coke samples; (**c**) The *Lc* and *La* values of different coke samples; (**d**) The *d*_(002)_ and *r*_0_ values of different coke samples.

Figure 4 provides the measured values of XRD parameters, e.g., the FWTH, 2 theta, *La*, *Lc*, *d*_002_, and *r*_0_ values of cokes estimated from the (002) carbon peak of cokes indicated in Fig. 3.

When compared to the original coke, the heat treatment temperature gradually decreases the interlayer spacing *d*_002_ and increases the *Lc* as well as the crystallinity of coke samples. It may be noted that the *Lc* values of cokes at different heat treatment temperature are greater than the original coke, demonstrating a higher degree of graphitization. Moreover, the *Lc* values increase as the heat treatment temperature rising, indicating an increased degree of ordering of carbon structure as well as graphitization. According to the relationship between the average stacking height (*Lc*) and the temperature, the XRD parameters suggested that the increase of the graphitization degree (*r*_0_) of coke may be related to an increasing temperature.

### Characterization of wetting behavior

**Figure 5:**
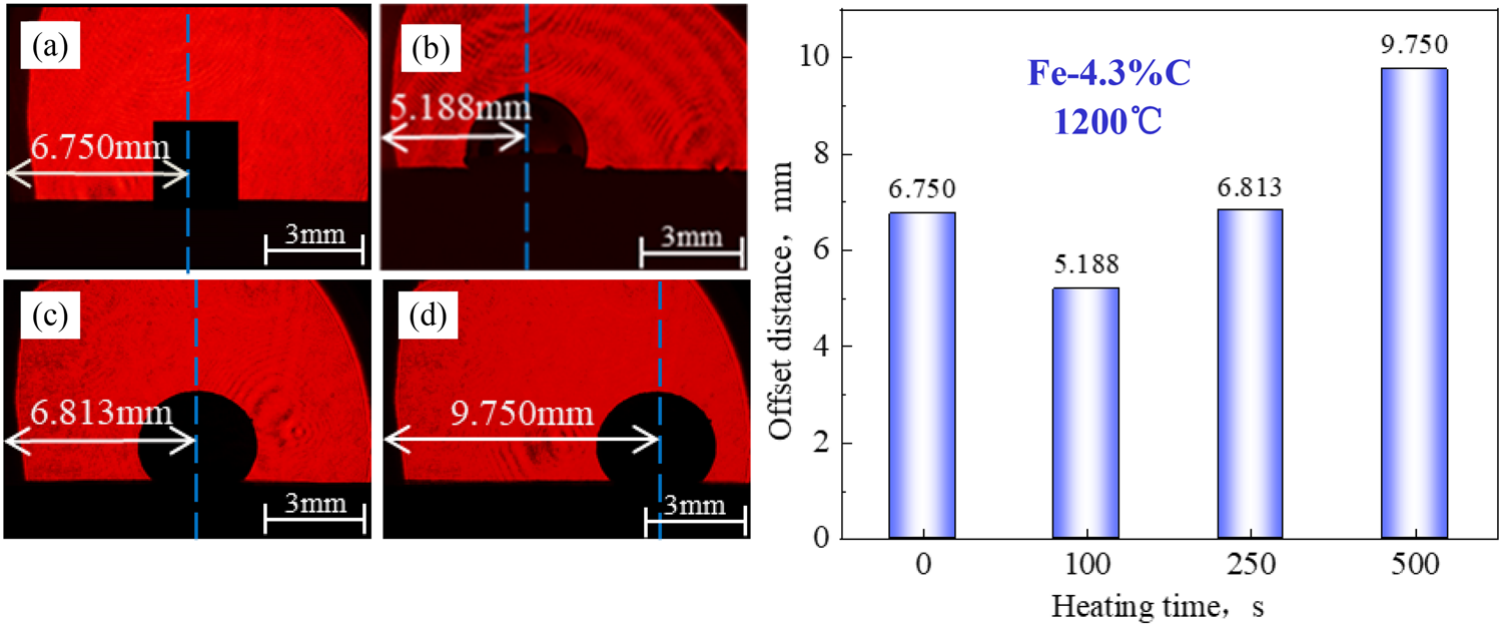
Wetting test of Fe–C melt on coke substrate heat-treated at 1200°C: (**a**) 0 s, (**b**) 100 s, (**c**) 250 s, (**d**) 500s.

**Figure 6:**
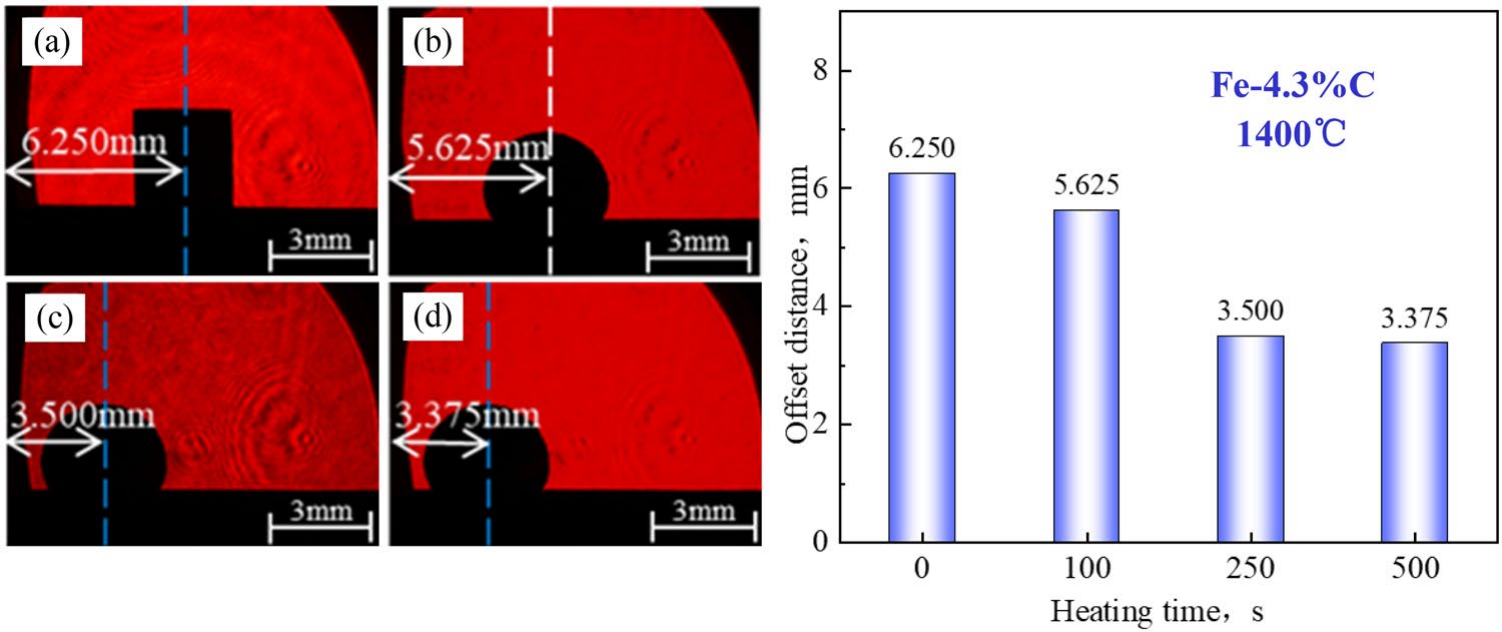
Wetting test of Fe–C melt on coke substrate heat-treated at 1400°C: (**a**) 0 s, (**b**) 100 s, (**c**) 250 s, (**d**) 500s.

**Figure 7:**
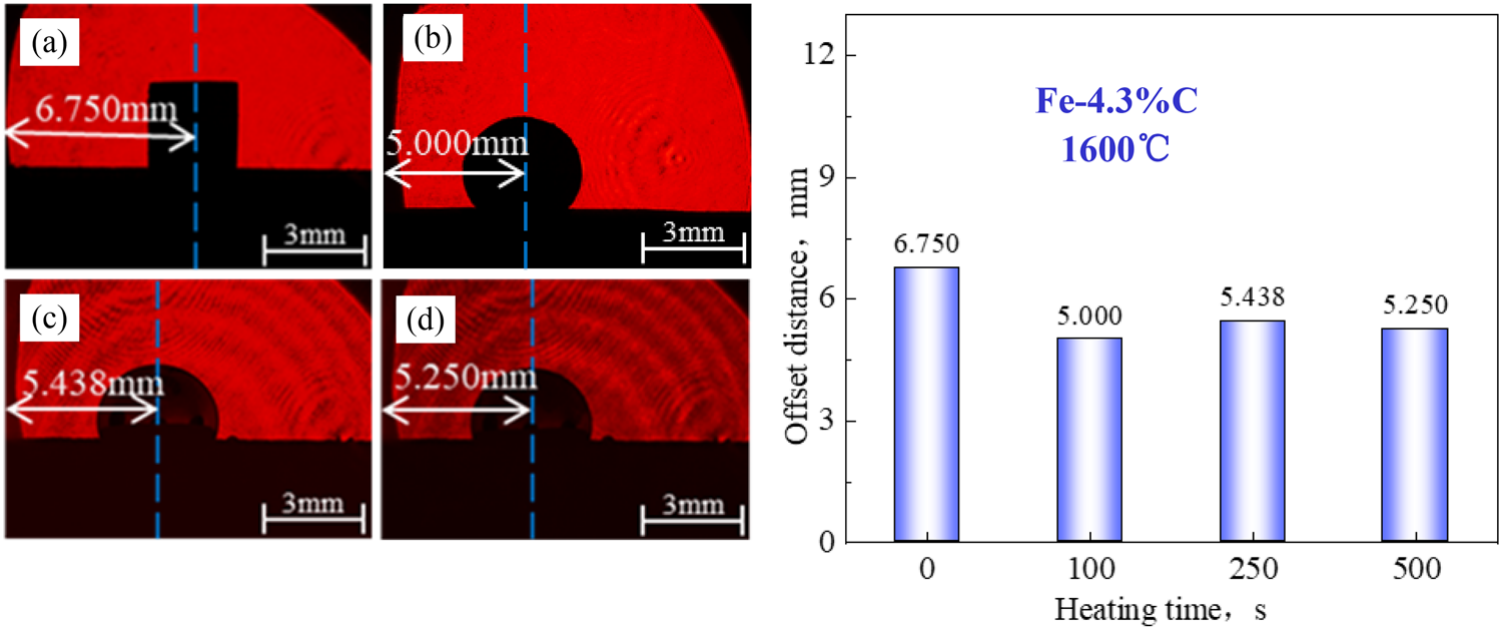
Wetting test of Fe–C melt on coke substrate heat-treated at 1600°C: (**a**) 0 s, (**b**) 100 s, (**c**) 250 s, (**d**) 500 s.

Figures 5, 6 and 7 show the wetting tests of Fe–C melt on coke substrates with varying *r*_0_.

In general, the Fe–C melt sample retains a cubic appearance at the start of the wetting experiment (t = 0 s); however, as time proceeds, the sample melts and transforms from a cube to a hemisphere.

The coke substrate with a low *r*_0_ has a large contact angle and is always nonwetting. The contact angle changes little over time (almost unchanged), and there is no wetting and spreading phenomenon. The Fe–C melt irregularly moved on the surface of the substrate after it melted into a hemisphere.

**Figure 8:**
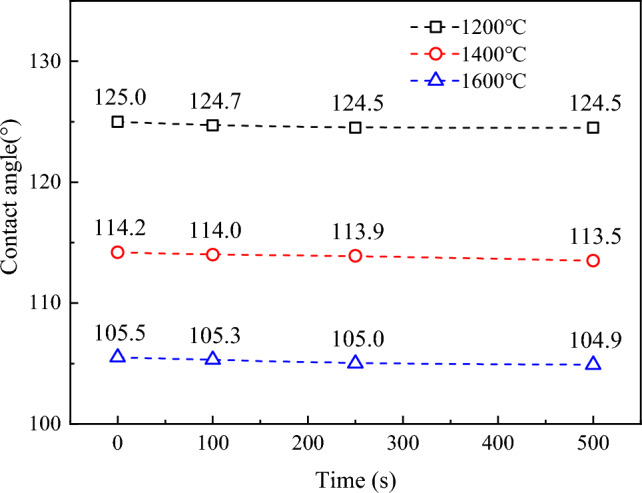
Curve of contact angle of Fe–C melt and coke with varying *r*_0_ with time.

Figure 8 shows the wetting curve of the contact angle of Fe–C melt on the coke substrate.

The Fe–C melt changes from solid to liquid during the melting process, causing morphological changes. The contact angle rarely changes during this period. The contact angles are 114.2°, 127.3°, and 136.7°, all of which are > 90°, thus indicating a nonwetting state.

Increasing the *L*c of the carbon structure crystallites can increase the dissolution rate, and the carbon crystal structure approaches a more ordered graphite. The higher the *r*_0_ of the carbon structure is, the lower is the bond energy between its atoms, and the easier it is for carbon atoms to dissociate from the matrix^26^. The wetting experiments show that increasing the *r*_0_ of the coke matrix material improves the wettability of molten iron, increases the contact area of the Fe–C interface, and improves carbon mass transfer, all of which are favorable to the carbon dissolution reaction.

### Interface micromorphology

**Figure 9:**
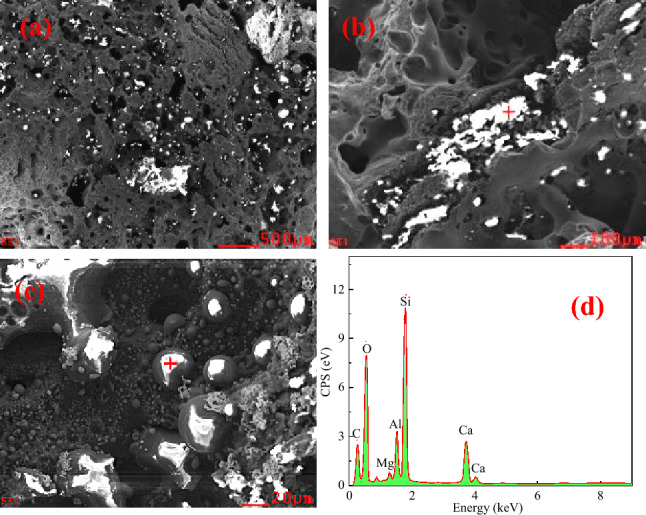
Micromorphology of interface between Fe–C melt and coke heat-treated at 1200℃. (**a**) interface shape with 200 times magnification; (**b**) interface shape with 1000 times magnification; (**c**) interface shape with 5000 times magnification; (**d**) EDS component analysis.

**Figure 10:**
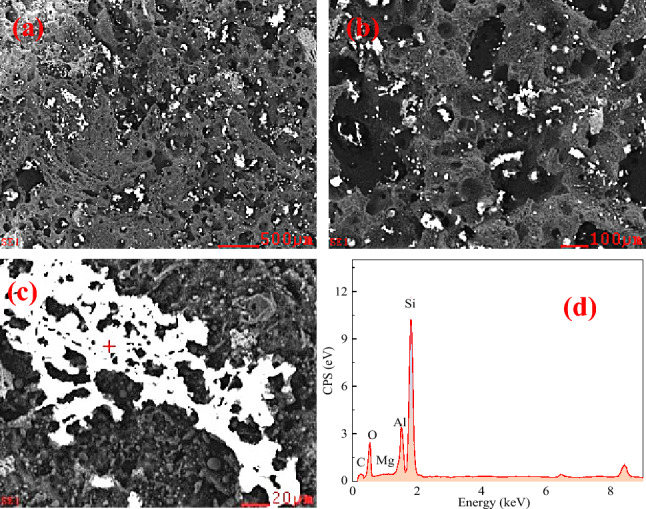
Micromorphology of interface between Fe–C melt and coke heat-treated at 1400℃. (**a**) interface shape with 200 times magnification; (**b**) interface shape with 1000 times magnification; (**c**) interface shape with 5000 times magnification; (**d**) EDS component analysis.

**Figure 11:**
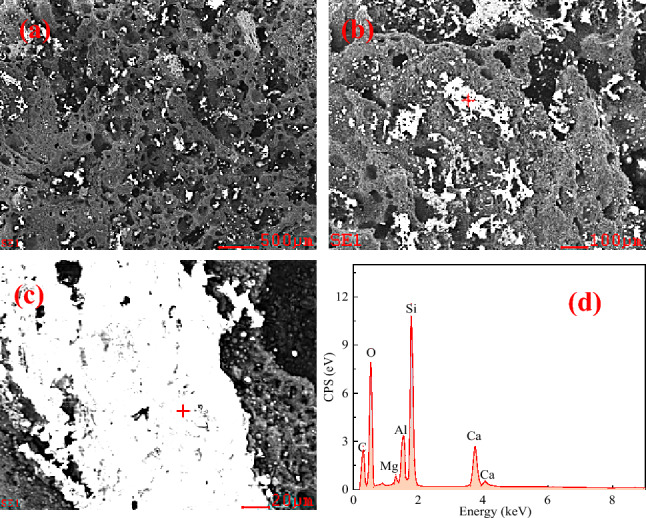
Micromorphology of interface between Fe–C melt and coke heat-treated at 1600℃. (**a**) interface shape with 200 times magnification; (**b**) interface shape with 1000 times magnification; (**c**) interface shape with 5000 times magnification; (**d**) EDS component analysis.

Because the Fe–C melt has a large contact angle with coke substrates with varying *r*_0_ and the interface contact area is small, the sample and substrate are not well wetted and bonded in the wetting tests. As shown in Figs. 9, 10 and 11, the morphology of contact interface at the position where the Fe–C melt falls off was examined using SEM and EDS analysis.

Before the experiment, the coke substrate was polished with sandpaper and it was smoothened and flattened. Following the wetting experiment, the substrate surface became rough and porous with irregular pits attached with bright white substances. EDS analysis demonstrated they are composed of Ca, Mg, Al, Si, and O, with the Si content being relatively high, indicating that they are Si-rich ash. The ash comes from the mineral matter transformation at high temperature. Typically, minerals in coke have a mass fraction of < 15% and are distributed in three ways: discrete distribution, dispersed distribution, and pore inclusions. The silicoaluminate is the most stable at high temperatures. When coke undergoes complex reactions, minerals, such as ash, will attach to the surface and enwrap the coke, thus forming a physical barrier and blocking the interface, reducing the contact area of the reaction between the coke and other objects. Because the melting point of these minerals is higher than the coking temperature, these minerals have not gone through the intermediate stage of melting. The carbon dissolution during the carburizing process causes these minerals to accumulate gradually in the interfacial region between coke and Fe–C melt, effectively reducing the available contact area between the two. Figures 9, 10 and 11 also indicate that the mineral layer is interspersed with large alumina or calcium alumina agglomerates. Besides, the agglomerates are partially more angular as Fig. 9 presents, suggesting a softening or melting of small particulates at high temperature. An increase in the graphitization degree is expected to form a thicker mineral matter layer. Highly ordered carbon structure generally improve carbon dissolution rate compared to less ordered forms of carbon. As a result, the morphology of the mineral layer is observed to change with graphitization degree, transforming from initially loose agglomerates to a dense layer.

### Analysis of the droplet movement

From Figs. 9, 10 and 11, it can be seen that the Fe–C melt droplets have varying degrees of left-to-right movement. The coke in the lower part of the blast furnace hearth is considered to be the only solid material, which supports the charged burdens and guarantees the permeability of the blast furnace, which plays an irreplaceable role in blast furnace production. The most important performance of coke in the lower regions in blast furnace hearth is carbon dissolution reaction with hot metal. If the dissolution reaction of coke with hot metal is easier to occur, the content of carbon in the hot metal is higher and carbon brick erosion is much more difficult. So it is better to guarantee the safety of the hearth refractory. Therefore, the disolution of coke and other carbon materials into liquid iron is an important step in the BF ironmaking process. Under liquid conditions, the contact reaction conditions of molten iron and coke are greatly improved. The coke is dissolved in the molten iron, and the carburization rapidly proceeds.

Based on this, the behavior of carbon as a surface-active element influences the movement of the droplet. Particularly, the driving force of droplet movement is deduced as follows:

1. The difference in interfacial tension between the left and right sides of the droplet

**Figure 12:**
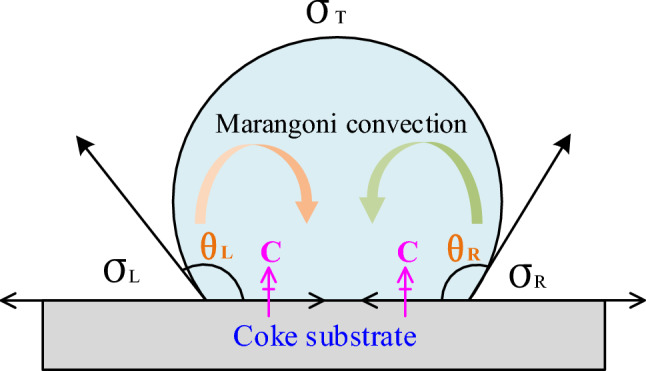
Schematic of Fe–C melt droplet interfacial tension and Marangoni convection.

Carbon was supplied from the droplet’s bottom in this study; however, the carbon distribution is asymmetric and different. As shown in Fig. 12, if the carbon concentration on the left is higher than that on the right, the interfacial tension (σ_L_) on the left is less than that on the right (σ_R_). The net horizontal force is determined by the interfacial tension on both sides and the contact angle (θ_L_ and θ_R_). This horizontal force could be the driving force for the droplet movement.

(2) Marangoni convection caused by a gradient in carbon concentration between the droplet's bottom and top

Carbon dissolves and penetrates the liquid iron from the coke substrate during the wetting experiment, i.e., the carbon concentration at the droplet's bottom is higher than the carbon concentration at the droplet’s top, which cause the interfacial tension on the top of droplet (σ_T_) as shown in Fig. 12. In hydrodynamics, the surface (or interfacial) tension difference or gradient is called the Maragoni convection^29,30^. Motions of liquid induced by the Marangoni effect, which is cailed Marangoni flow or Marangoni convection, are most intensive at the surface or the interface. There the motion can effectively promote both the masstransport and heat transport across the interface. Liquid metals and slags generally have high surface or interfacial tension and also have strong surface active components such as oxygen and sulfur in liquid iron.

Consequently, the Marangoni convection will move from the bottom with low interfacial tension to the top with high interfacial tension. Downward convection will form in the central part of the droplet because of the Marangoni convection and will circulate until the interfacial tension gradient disappears. Owing to the asymmetric distribution of bottom carbon in the experiment, asymmetric Marangoni convection will form in the droplet, and the droplet will move via the asymmetric convection.

(3) Interfacial phenomenon at the interface layer

Figures 9, 10 and 11 show that there are many ash materials with high SiO_2_ content at the interface illustrated by SEM–EDS analysis. During the vacuum carbothermal reduction process, SiO_2_ is known to be volatile. The coke in BF is used as a carbon source for carburizing and in situ SiO_2_ reduction, which consumes some amount of carbon. Therefore, at 1450 °C, 5 × 10^−4^ Pa vacuum conditions, there will be the following reactions:

4 $$ {\text{SiO}}_{{2}} \left( {\text{s}} \right) \, + {\text{ 3C}}\left( {\text{s}} \right) \, = {\text{ SiC}}\left( {\text{s}} \right) \, + {\text{ 2CO}}\left( {\text{g}} \right) \, \Delta G_{T} = { 59495}0 \, - { 523}.{8}0{58}T + { 16}.{628}T{\text{ln}}p $$

5 $$ {\text{SiO}}_{{2}} \left( {\text{s}} \right) \, + {\text{ C}}\left( {\text{s}} \right) \, = {\text{ SiO}}\left( {\text{g}} \right) \, + {\text{ CO}}\left( {\text{g}} \right) \, \Delta G_{T} = { 675842}.{6 } - { 55}0.{422}T + { 16}.{628}T{\text{ln}}p $$

**Figure 13:**
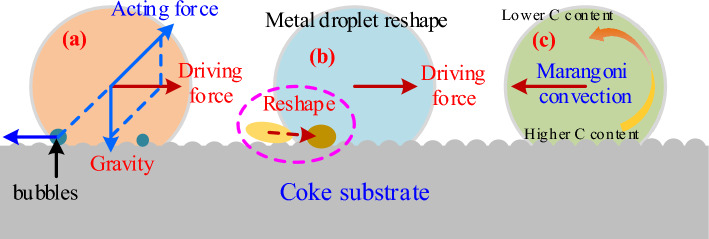
Schematic of irregular movement of Fe–C melt.

The generation, aggregation, and release of SiO bubbles may influence the irregular movement of Fe–C melt. Figure 13 shows a schematic of Fe–C melt movement on the coke substrate.

The ‘irregular movement of Fe–C melt’ pheonomenon strongly depends on the coupling force of the net horizontal force, the Marangoni convection, and the offset pressure caused by the generation, aggregation and release of SiO bubbles at the interface. During carbon dissolution process at high temperature, small SiO bubbles *in-situ* form at the interface between the Fe–C melt and coke due to the reaction (5). The quantity of SiO bubbles increases with extension of test time. Small bubbles join together to form larger bubbles to reduce the surface energy and achieve stability. Because the vertical distance between the interface and melt surface is not constant, the static pressure of bubbles will vary at different points along with the interface. The static pressure of the bubble at the interface’s center is greater than the static pressure on both sides of the interface, causing the bubbles to have offset pressure to both sides. In other words, the offset pressure from the center of the alloy droplet to both sides drives the bubble to the outside of the melt, causing the Fe–C melt to move in the opposite direction. Besides, with the dissolution reaction of coke with Fe–C melt proceeding, the concentration gradient of carbon tends to be increased. As a result, the Maragoni convection and the aforementioned processes (generation, aggregation, and release of SiO bubbles) repeat, causing irregular movement of the Fe–C melt droplets.

In general, it is difficult to estimate the contribution of each driving force from the experiment, including the net horizontal force caused by the difference in interfacial tension and contact angle, the asymmetric Marangoni convection caused by the carbon's bottom and top concentration gradient, the droplet profile, and the offset pressure caused by the generation, aggregation and release of SiO bubbles at the interface. However, the droplet movement will be determined by these three factors, which may independently occur or together.

## Conclusion

1. As the heat treatment temperature increases, the *d*_002_ of the graphite crystallites in the coke gradually decreases, the *L*c of coke increases, and *r*_0_ deepens. During the carbon dissolution process on the coke substrate with different *r*_0_, the Fe–C melt does not wet and spread. The contact angles are 124.5°, 113.9°, and 105.3°, indicating a nonwetting state. The deepening of graphitization improves wettability, increasing the contact area between liquid iron and carbonaceous material, promoting the carbon dissolution.
2. Under high temperatures, a portion of carbon particles fell off the surface of the coke matrix, forming irregular pits that appeared porous and attached more amount of bright white Si-rich ash and surface roughness of the matrix considerably increased. The wetting and spreading behaviors of droplets of both sides are strongly hindered, resulting in non-wetting and spreading. The contact area between the Fe–C melt droplets and carbon matrix material is greatly reduced, resulting in weakened carburization.
3. The driving force for the irregular movement of the Fe–C melt droplets is the net horizontal force determined by the difference in interfacial tension and contact angle caused by uneven carbon distribution, the asymmetric Marangoni convection caused by the bottom and top concentration gradients of the carbon as well as the drop profile, and the offset pressure caused by the generation, aggregation, and release of SiO bubbles at the interface.
4. The graphitization of coke with higher ording of carbon structure could accelerate the carbon dissolution process, which prevents circuiting flow of hot metal and reduces the contact area between the iron slag and the refractory bricks, resulting in the reduction of corrosion rate. It is of particle importance to investigate the contribution of each force, e.g. the interfacial tension, the Marangoni convection, and SiO bubbles generation in future study.

## Data Availability

Due to the nature of this research, participants of this study did not agree for their data to be shared publicly, so supporting data is not available. If you would like to obtain data from this study, please contact the corresponding author (Dr. Zhan) at zhanwenlong@ustl.edu.cn.
